# Role of Co in the Electrocatalytic Activity of Monolayer Ternary NiFeCo-Double Hydroxide Nanosheets for Oxygen Evolution Reaction

**DOI:** 10.3390/ma14010207

**Published:** 2021-01-04

**Authors:** Ye Li, Dan Zhao, Yue Shi, Zhicheng Sun, Ruping Liu

**Affiliations:** 1 Beijing Institute of Graphic Communication, School of New Media, Beijing 102600, China; liye@bigc.edu.cn; 2 Beijing Institute of Graphic Communication, School of Printing and Packaging Engineering, Beijing 102600, China; zhaodan_1997@163.com (D.Z.); shiyuematerials@gmail.com (Y.S.); sunzhicheng@bigc.edu.cn (Z.S.)

**Keywords:** monolayer nanosheets, defect, water splitting, oxygen evolution

## Abstract

Monolayer nanosheets have gained significant attention as functional materials and also in photo/electrocatalysis due to their unique physical/chemical properties, abundance of highly exposed coordination sites, edges, and corner sites, motivating the pursuit of highly active monolayer nanosheets. NiFe-based layered double hydroxide (NiFe-LDH) nanosheets have been regarded as the most efficient electrocatalysis for oxygen evolution. However, the limited catalytic active site and the stacking layer limited the performance. Therefore, by introducing highly electroactive Co ions into monolayer NiFe-LDH, the obtained ternary NiFeCo-LDH monolayer structure possessed an increased concentration of defect (oxygen and metal vacancies), providing enough unsaturated coordination sites, benefitting the electrocatalytic water oxidation, as also explained by the density functional theory (DFT). This work reported an efficient strategy for the synthesis of ternary monolayer LDH in the application of energy conversion and storage.

## 1. Introduction

Electrochemical water, which is created by splitting H_2_ and O_2_, has been regarded as a clean and renewable energy source for the next generation, owing to its zero-carbon emission, high abundance, and high energy conversion efficiency in renewable energy fields [1,2]. However, due to the half-reaction of water splitting, the oxygen evolution reaction (OER) greatly limits the performance of the water splitting due to its sluggish kinetic feature. To address this bottleneck problem, designing and fabricating a novel OER catalyst has been a research hotspot [3]. Currently, the state-of-the-art electrocatalysts are normally focused on noble metal oxides (such as IrO_2_ and RuO_2_). Nevertheless, it is impractical to use them in large-scale experiments because of the high price and scarcity. Therefore, earth-abundant electrocatalysts with inexpensive, high efficiency, and well stability are highly desirable [4]. Among the alternative electrocatalysts, compounds of the transition metals (Co, Ni, Fe, Mn, V, etc.) display great promises as competent electrocatalysts for OER and have gained much attention because of their earth-abundant and well catalytic performance in alkaline solution [5].

In previous studies, researches have demonstrated that the electrochemical properties of the materials were structure-dependent, relating to their performance [6]. Designing nanosized and/or nanostructured materials is an effective way to improve their performance [7,8,9]. Among them, monolayer 2D nanomaterials, which possess just single atomic layers in the c axis, have gained much attention since the discovery of the isolation of graphene [10]. These atomic thin nanosheets, owing to their special physical and chemical properties, have widely displayed potential applications in fields ranging from electronics to catalysis, and so on [11]. Based on theoretical and experimental studies, it has been demonstrated that the enlarged surface area and vast atomic defects in monolayer nanomaterials can promote the mobility efficiency of the surface carriers (such as electrons or photo-induced holes) and then increase the corresponding catalysis [12]. Taking MoS_2_ as an example, the electrocatalytic overpotential of the MoS_2_ monolayer significantly reduced over 100 mV, with the catalytic current being enhanced several times, compared with that of the bulk MoS_2_ [13,14]. Compared with other materials, transition metal-based hydrotalcite (also known as layered double hydroxide (LDH)), which is a type of two-dimensional (2D) layered clays [15], has been regarded as one of the best candidates for OER catalysts [16] because of its wide compositions of LDHs, which can be fabricated by adjusting the metal species and ratios in the layer and interlayer spacing [17]. 3D NiFe-LDH film growth on Ni foam showed improved electrocatalytic performance [4]. Various ternary NiFeMn [18], NiFeV [19], NiFeCr-LDH, ultrathin NiCoFe-LDH, and Au/NiFe-LDH [20,21,22,23,24] nanosheets with a thickness of more than 4 nm also demonstrated improved electrooxidation performance [25]. The introduction of some electronically active species (such as Co, Mn, etc.) into NiFe-based structure would specifically benefit the water splitting performance, mainly due to the prompt effect on the fully exposed active sites, accelerated charge transfer/transport, etc. However, the LDH particles synthesized by the normal method were stacking seriously [26], resulting in a low specific surface area and undesirable poor conductivity, which significantly restricted the development in the electrocatalytic activity [27]. Recently, ultrathin and even monolayer LDH [11,28,29,30] structure, as first reported by O’Hare and Sasaki, have been gaining great attention [17]. This is mainly due to the highly exposed active sites with increased conductivity, which provide a robust platform for water splitting into oxygen [31,32], especially NiFe-LDH and NiTi-LDH [33,34,35,36,37]. However, few detailed investigations have been undertaken on how to further enhance the OER activity of monolayer NiFe-based LDH, which has limited the development of the monolayer LDH-based OER catalyst.

In this work, by introducing Co species into the monolayer NiFe-LDH, the monolayer NiFeCo-LDH nanosheets have been fabricated as excellent electrocatalysts in electrochemical water splitting. The thickness of NiCoFe-LDH nanosheets was determined to be 1 nm, corresponding to the monolayer, which can greatly enhance the surface area as well as provide a lot of exposed edges and defects, as evidenced by X-ray absorption fine spectra, leading to optimized electrochemical reaction and the transportation of electrons. Moreover, density functional theory (DFT) calculations were used to investigate the geometric and electronic structures of the monolayer NiFeCo-LDH nanosheets in order to explain the relationship between material composition and electrocatalytic performance in detail.

## 2. Materials and Methods

### 2.1. Materials

Chemical reagents, including Ni(NO_3_)_2_·6H_2_O, Fe(NO)_3_·9H_2_O, Co(NO_3_)_2_·6H_2_O, Zn(NO_3_)_2_·6H_2_O, Al(NO_3_)_3_·6H_2_O, NaOH, and urea, were of analytical grade and were used without further purification. Formamide was obtained from Sigma-Aldrich Co (Saint Louis, MO, USA). De-ionised and decarbonated water was used in all experiments.

### 2.2. Synthesis of Monolayer NiFe-LDH Nanosheets (Denoted as NiFe-Mono)

A 20 mL aqueous solution containing 33.4 mM Ni(NO_3_)_2_ × 6H_2_O and 16.7 mM Fe(NO_3_)_3_ × 9H_2_O was added dropwise to 20 mL of a 20 vol% formamide solution under magnetic stirring at 80 °C [38,39]. Simultaneously, 0.25 M NaOH was gently dropped into the above solution to maintain a pH of ~10 for the formation of LDH structure. The reaction was completed within around 10 min, the LDH structure was formed in the meantime. Then, the product was treated by centrifugation, washed with a mixture of ethanol and water (1:1 by volume) several times to move the extra absorbed species (such as formamide, etc.), and the monolayer NiFe-LDH nanosheets were kept in the wet state for subsequent use after centrifugation.

### 2.3. Synthesis of Monolayer Co-Containing NiFeCo-LDH Nanosheets (Denoted as NiFeCo-Mono)

Co(NO_3_)_2_ × 6H_2_O (20% mole ratio, compared with Ni) was added into the solution as precursor salt for the synthesis of Co-containing NiFeCo-LDH monolayer. The synthesis of the method is the same as the above synthesis of NiFe.

## 3. Results and Discussion

### 3.1. Synthesis and Morphology Characterization of Monolayer NiFeCo-LDH Nanosheets

**Scheme 1:**
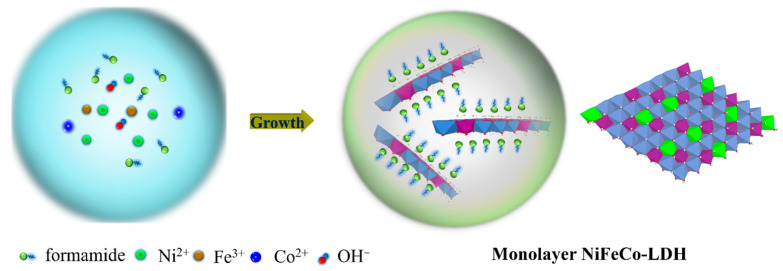
The synthetic strategy used for the synthesis of monolayer Co-containing NiFe-LDH nanosheets.

**Figure 1:**
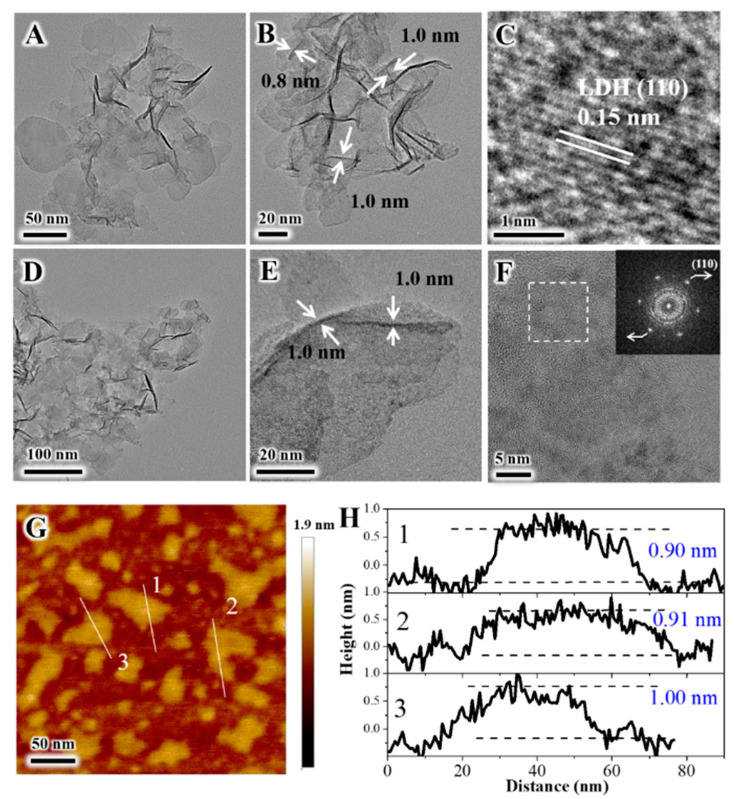
(**A**,**B**) TEM and (**C**) HRTEM image of NiFe-mono; (**D**,**E**) TEM images of NiFeCo-mono, respectively; (**F**) HRTEM of NiFeCo-mono; the inset is the corresponding FFT pattern. (**G**) AFM image and (**H**) height profiles of NiFeCo-mono. The numbers 1 to 3 in (**H**) correspond to the numbers 1 to 3 in (**G**).

The monolayer NiFe-LDH and the NiFeCo-mono nanosheets were obtained by an in situ growth process when introducing formamide as layer growth. The synthesis process is illustrated in Scheme 1. The typical morphology and structures of the monolayer NiFeCo-LDH nanosheets and NiFe-mono were investigated in detail. As shown in Figure 1A,B, the monolayer NiFe-LDH nanosheets gave a thickness of ~1.0 nm with the size of 40 nm, of which the thickness is near to the reported monolayer MgAl-LDH nanosheets [40]. High-Resolution Transmission Electron Microscopy (HRTEM) in Figure 1C also shows the space of the lattice of 0.15 nm, assigned to be the (110) facet of monolayer LDH [31]. The Atomic Force Microscopy (AFM) image also gave the evidence for the successful synthesis of LDH monolayer with a thickness of 1.0 nm [11]. By adding Co ions into monolayer NiFe-LDH, based on the TEM and HRTEM images in Figure 1D–F, the monolayer NiFeCo-LDH (NiFeCo-mono) nanosheets exhibited a plate-like shape with a mean thickness of 1.0 ± 0.1 nm. The high-resolution TEM (HRTEM) image of NiFeCo-mono nanosheets also demonstrated the lower crystalline degree, with the FFT pattern of hexagon, further giving evidence for the LDH structure. X-ray Powder Diffraction (XRD) also provided the evidence for the successfully synthesis of monolayer NiFeCo-mono structure (Figure S1). Furthermore, the thickness of monolayer NiFeCo-mono nanosheets was further determined by AFM to be about 0.9 ± 0.1 nm. All of the above results illustrated that atomically thin NiFeCo-mono nanosheets have been successfully synthesized. The monolayer structures with all atoms exposed could provide numerous electroactive sites and may affect the reaction kinetics, which will be discussed further.

### 3.2. Electrocatalytic Water Splitting by Monolayer NiFeCo-LDH

**Figure 2:**
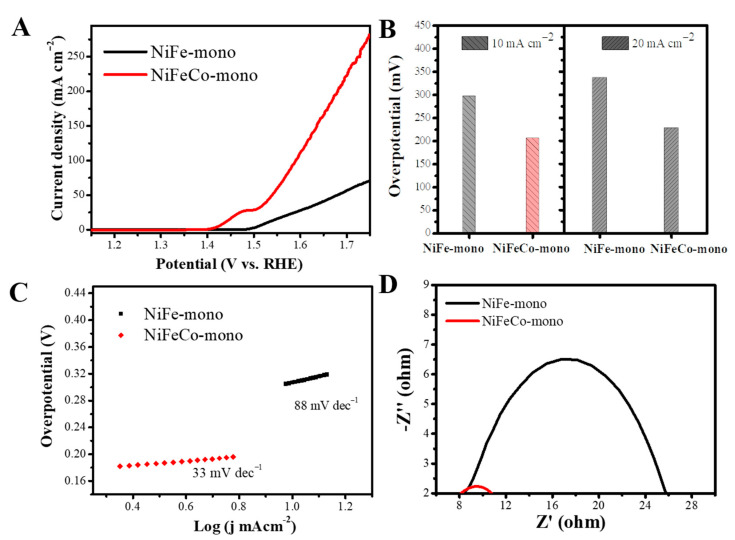
OER polarization curves (**A**) and their overpotential (**B**) for NiFe-mono and NiFeCo-mono, respectively, at 10 × mA cm^−2^ and 20 mA × cm^−2^; sweep rate: 5 mV × s^−1^; (**C**) Tafel plots (RHE vs. log current) derived from (**A**,**D**) Electrochemical impedance spectra for NiFe-mono, NiFeCo-mono, respectively.

The electrocatalytic performances of the monolayer NiFeCo-LDH were investigated by linear sweep voltammetry (LSV) measurement to perform the OER catalytic activity in 1 M KOH aqueous. For comparison, the OER performance of monolayer NiFe-LDH was also evaluated simultaneously. As shown in Figure 2A, two oxidation waves can be observed at around 1.425 V and 1.5 V vs. RHE over monolayer NiFeCo-mono, which can be explained by the first supercapacitor observed from Co^2+^ to Co^3+^ with the following oxidation process from Co^3+^ to Co^4+^, etc. Similar oxidation waves in NiCoFe-based LDH and oxides have been reported previously [24]. Compared with the LSV polarization curve of NiFe-LDH, the monolayer NiFeCo-mono display a slightly low onset potential and higher oxygen-evolving current, indicating that Co doping had a significant effect in altering the electrocatalytic OER performances. Furthermore, the overpotential of the OER catalyst at the current density of 10 mA cm^−2^ and 20 mA cm^−2^ was required, since it is a crucial parameter for the OER catalyst and was widely used to evaluate the catalytic activity. The overpotential of all the materials at 10 mA cm^−2^ and 20 mA cm^−2^ was shown in Figure 2B. As for the monolayer NiCoFe-mono, the overpotential corresponding at a current density of 10 mA cm^−2^ was 297 mV (NiFe-mono), 206 mV (NiFeCo-mono), respectively. It is obvious to see that when introducing Co species in the monolayer NiFeCo-mono, the overpotential is significantly lower than that of monolayer NiFe-mono, displaying the optimized lower overpotential at the same condition, further confirming that the effect of doped Co into the monolayer NiFe-LDH is beneficial to improve the electrocatalyst performance. A similar phenomenon can also be obtained at the current density of 20 mA cm^−2^ or higher. Such lower overpotential was much lower than similar materials as mainly due to the effect of Co-intercalation and also the formation of defect, as discussed below. Meanwhile, the Tafel plots of the monolayer NiCoFe-mono and NiFe-mono content were investigated and presented in Figure 2C. The Tafel plot is another important kinetic factor to evaluate for the electrochemical OER performance of the electro material, since it can display the rate of OH^−^ at a low overpotential range in the rearrangement and deprotonation process for OER. As can be seen in Figure 2C, the Tafel slope decreased from 88 mV per decade to 33 mV per decade when introducing Co in NiFe-mono, which is in accordance with the evolution of the overpotential at the current density of 10 mA cm^−2^, suggesting that the cobalt incorporation in NiFe-LDH enabled faster deprotonation of OH^−^ and is more facile for the electron transport in the OER process, which can be attributed to the introduction of cobalt into NiFe-LDH, which provides additional active sites for OH^−^ adsorption, as well as promoting the electric conductivity. To further investigate the corresponding kinetic activity, the electrochemical impedance spectroscopy (EIS) was explored further (Figure 2D). The Nyquist plots displayed that the semicircle diameter of NiFeCo- mono is much lower, which equals the interface charge transfer resistance, demonstrating the lowest resistance and the fastest charge transfer process for the NiFeCo-mono during the OER process among all materials herein.

Based on the dates of the experiments above, it can be concluded that the monolayer NiFeCo-mono can exhibit optimized OER performance compared with NiFe-mono; therefore, the monolayer NiFeCo-mono nanosheets were chosen as the research target, as following in this work. The durability test is critical for OER catalysts in the energy systems. Figure S2A displayed the stability of NiFeCo-mono by means of the chronopotentiometry method for more than 8 h both at 1.436 V and 1.540 V vs. RHE. Moreover, NiFeCo-mono gave about a ~100% Faradaic yield, indicating the O_2_ evolution was driven by NiFeCo-mono electrocatalyst (Figure S2B,D). The steady current response reveals the high stability of the monolayer NiFeCo-mono nanosheets.

### 3.3. Discussion on the Relationship between the Structure and the Performance of NiFeCo-Mono

**Figure 3:**
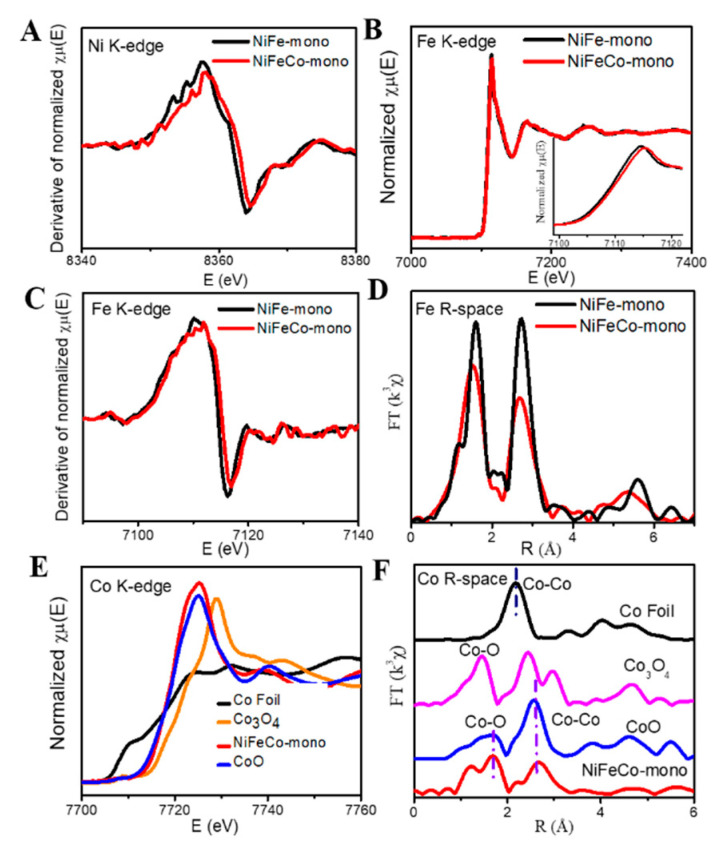
(**A**) The first derivative of Ni K-edge X-ray absorption near-edge structure (XANES) spectra; (**B**) Fe K-edge XANES spectra and (**C**) the first derivative; (**D**) magnitude of k^3^-weighted Fourier transforms of the Fe K-edge XANES spectra for NiFe-mono and NiFeCo-mono; (**E**) Co K-edge XANES spectra and (**F**) magnitude of k^3^-weighted Fourier transforms of the Co K-edge XANES spectra for NiFeCo-mono and the reference CoO, Co_3_O_4_ and Co foil samples.

The effect of the Co ions in monolayer NiFeCo-mono has also been explored by using X-ray absorption near-edge structure (XANES) spectroscopy. As shown in Figure 3A, the first derivative of Ni K-edge of NiCoFe-mono shifts to higher energy, indicating the higher oxidation state of Ni in NiFeCo-mono, compared with mono NiFe-LDH. As shown in Fe K-edge XANES given in Figure 3B,C, the oxidation of Fe in NiFeCo-mono slight moved higher, further demonstrating that the additive of Co in the mono NiFe-LDH results in a higher valence state of Ni and Fe ions in NiFeCo-mono. The reason can also be explained from the corresponding R space plot of Fe K-edge. As shown in Figure 3D, the coordination number (*N*) of the first shell (Fe–O) and the second shell (Fe–Fe/Ni) for monolayer NiFe-mono around 1.5 and 3.0 Å give the numbers of 5.7 and 5.5 (Table S1). More interestingly, after adding Co ions into mono NiFe-mono, the Fe–O shell for the NiFeCo-mono had a lower intensity, with a lower N (5.4), further indicating the existence of the higher abundance of oxygen vacancies (V_O_) with severe structural distortion appearing in the NiFeCo-mono. Furthermore, in the second Fe–Ni/Fe shell, N also decreased to 4.7 for NiFeCo-mono from 5.3 for mono NiFe-mono, demonstrating an increased formation of metal vacancies (V_Ni_ and V_Fe_) with the severe distortion and the lower crystal degree. This result was in accordance with the HRTEM image, explaining the lower crystal degree with more defect of NiFeCo-mono. As shown in Figure 2E,F, Co K-edge of NiFeCo-mono also gives a similar adsorption spectra and structure to CoO structure, with a +2 oxidation state. The chemical coordinative environment of Co is similar to that of Fe in NiFeCo-mono (Figure 3D,F), further indicating that Co intercalation in NiFeCo-mono happens at the Fe site, not interstitially.

**Figure 4:**
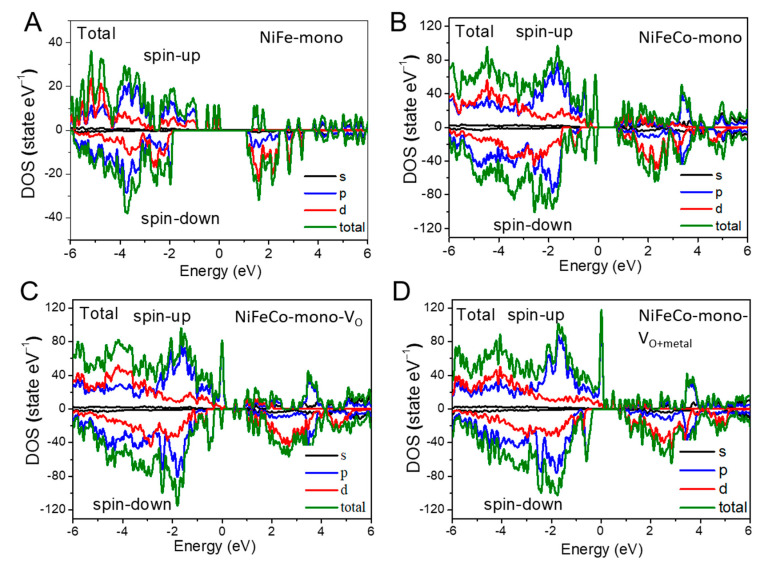
The calculated total DOS/partial DOS (PDOS) plots for (**A**) NiFe-mono; (**B**) NiFeCo-mono; (**C**) NiFeCo-mono-V_O_; (**D**) NiFeCo-mono-V_O+metal_.

**Figure 5:**
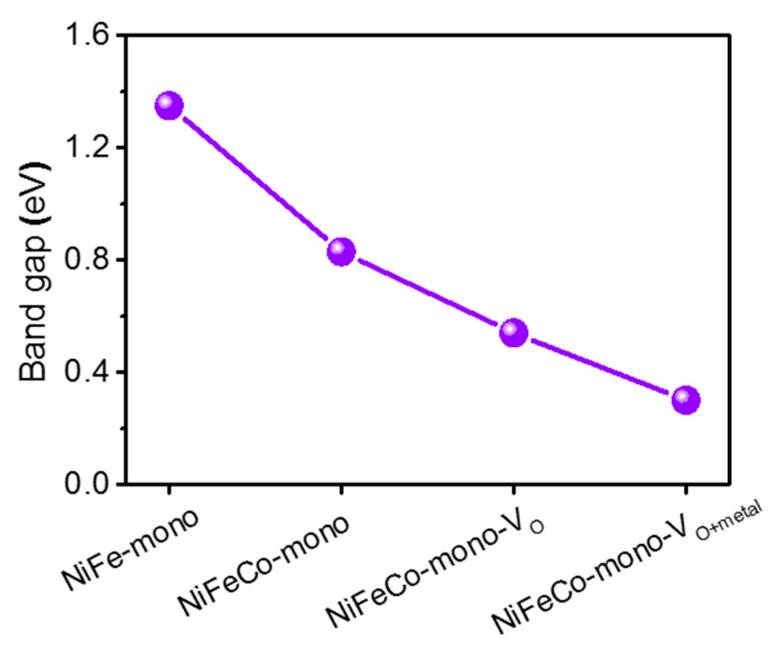
The calculated band gap of NiFe-mono, NiCoFe-mono, NiCoFe-mono-V_O,_ and NiCoFe-mono-V_O_+metal.

In order to understand the Co-doped effect in monolayer NiFe-LDH, and the defect role in NiFeCo-mono, DFT calculations were performed in detail. Structure models (Figure S3) were built based on pure monolayer NiFe-LDH (denoted as NiFe-mono), Co-doped mono-NiFe-LDH (NiFeCo-LDH), and defect (oxygen vacancies and metal vacancies) doped mono-NiFe-LDH (denoted as NiCoFe-mono-V_O_ and NiCoFe-mono-V_O+metal_). As shown in Figure 4, NiFe-mono had a bandgap of 1.35 eV, which is accordance with the reported value. After doping Co ions into NiFe-mono, the bandgap of NiCoFe-mono narrowed to 0.8 eV, mainly due to the existence of Co ions. Interestingly, when defects were induced into NiFeCo-mono, the DOS revealed that there was no obvious gap with, only 0.5 and 0.3 eV for NiCoFe-mono-V_O_ and NiCoFe-mono-V_O+metal_ (Figure 5), respectively, indicating that the defect effect contributed to the increased conductive properties of NiCoFe-mono, further confirming the semi-metal-like character. The above DFT results are in accordance with a good conductivity of the NiFeCo-mono, as given in EIS results, further implying a new perspective on the strategy of introducing Co—not only change to the electronic structure of NiFe-mono, but also to give another effect on the formation of a defect in the monolayer ternary LDH for the OER performance.

## 4. Conclusions

By introducing monolayer NiFe-mono nanosheets with Co species, the resulting monolayer NiFeCo-mono exhibited enhanced water oxidation ability with lower overpotential. EXAFS proved that the increased content of defect (oxygen and metal vacancies) in monolayer NiFeCo-mono is mainly due to the introduction of Co ions in monolayer NiFe-mono lattice, which further benefits the charge-carrier transformation kinetics. DFT calculations further indicated that the decreased band gap of the defect-containing monolayer NiCoFe-LDH was explained by the enhanced electrochemical activity. In summary, this work reported a route for enhancing monolayer binary NiFe-LDH activity by introducing the electroactive Co species, or, Cr, Mn, Cu ions, providing an approach for the rational design of ternary monolayer LDH for efficient energy conversion and storage.

## Data Availability

Data sharing not applicable.

## Supplementary Materials

The following are available online at https://www.mdpi.com/1996-1944/14/1/207/s1, Figure S1. XRD patterns of (A) the wet NiFeCo-mono colloid sample, and (B) the NiFeCo-mono after drying at 100 degree, respectively, Figure S2. I-t curve and the electrocatalytic efficiency of electrode loaded with NiFeCo-mono at (A, B) 1.436 V vs. RHE and at (C, D) 1.540 V vs. RHE, respectively. (The solid line in (B, D) means the theoretically expected O_2_, and the circle respends to the experimentally tested O_2_ from GC with TCD detector). Figure S3. The models of (A) NiFe-mono, (B) NiFeCo-mono, (C) NiFeCo-mono-V_O_ and (D) NiFeCo-mono-V_O+metal_, Table S1. Fitting results from the EAXFS data of NiFe-mono and NiFeCo-mono.
