# Supplementary material for: Role of Co in the Electrocatalytic Activity of Monolayer Ternary NiFeCo-Double Hydroxide Nanosheets for Oxygen Evolution Reaction
Source: Materials (Basel). 2021 Jan 4;14(1):207. doi: 10.3390/ma14010207 (PMC7795402; doi:10.3390/ma14010207)
Supplement: Supplementary file 1 [file materials-14-00207-s001.pdf]

materials

Supplementary Information

Role of Co in the Electrocatalytic Activity of Monolayer Ternary NiFeCo-Double Hydroxide Nanosheets for Oxygen Evolution Reaction

Ye Li [1], Dan Zhao [2], Yue Shi [2], Zhicheng Sun [2] and Ruping Liu [2,*]

[1] Beijing Institute of Graphic Communication, School of New Media, Beijing 102600, China; liye@bigc.edu.cn
[2] Beijing Institute of Graphic Communication, School of Printing and Packaging Engineering, Beijing 102600, China; zhaodan_1997@163.com (D.Z.); shiyuematerials@gmail.com (Y.S.); sunzhicheng@bigc.edu.cn (Z.S.)
* Correspondence: liuruping@bigc.edu.cn; Tel.: +86-010-6026-1603

Supplementary Materials: The following are available online at www.mdpi.com/xxx/s1.

S1.1.Characterization

The XRD was obtained on a Rigaku UltimaIII. JEOL JEM-2010 was used for collecting HRTEM images. XAFS measurements were obtained from the BL14W1 of Shanghai SSRF. The metal concentrations were measured by inductively coupled plasma (ICP) (ICP-6300, Thermo Fisher Scientific, USA).

S1.2. Electrochemical measurements

All electrochemical measurements were performed in using a CH Instruments (CHI 660E) work station with three-electrode system (counter electrode: Platinum (Pt) electrode, reference electrode: saturated calomel electrode (SCE)) in 1.0 M KOH aqueous solution. The reversible hydrogen electrode (RHE) $E_{vs\ RHE} = E_{vs\ SCE} + E_{SCE} + 0.059pH$). The working electrode was obtained by loading 100 μL ethanol dispersion solution containing monolayer LDHs (6 mg of catalyst was dispersed in 1 mL of ethanol followed by sonication for ~ 30 min) on 1×1 cm^2 carbon-fiber paper with the following dropping 10 μL of a 0.5 wt.% Nafion solution. The scan rate was 5 mV s^{-1} for LSV. The EIS was conducted over 1-10^5 Hz. The Faradic efficiency of the monolayer LDH was defined as the ratio of the evolution amount of O_2 as a test by TCD to that of the theoretically expected O_2 from the water oxidation process. The theoretical amount of O_2 was then calculated by applying the Faraday law (96485.4 C causes 1 equivalent of reaction), and four mol electrons equal to 1 mol O_2 during OER process.

S1.3. Computational details

DFT + U calculations were utilized using CASTEP module in Material Studio (The values of U – J (U_{eff}) was 3.87 eV for Ni, and 4.3 eV for Fe and 3.52 for Co) [1]. The GGA with PBE functional was employed and 340 eV of cutoff was assigned to the plane-wave basis set. The SCF tolerance was 1×10^{-6} eV with 1×1×1 k-points for Brillouin zone, and ultrasoft pseudo-potentials for the core electrons.

Citation: Li, Y.; Zhao, D.; Shi, Y.; Sun, Z.; Liu, R. Role of Co in the Electrocatalytic Activity of Monolayer Ternary NiFeCo-Double Hydroxide Nanosheets for Oxygen Evolution Reaction. *Materials* **2020**, *14*, x. https://doi.org/10.3390/xxxxx

Received: 22 October 2020
Accepted: 14 December 2020
Published:

Publisher's Note: MDPI stays neutral with regard to jurisdictional claims in published maps and institutional affiliations.

Copyright: © 2020 by the authors. Licensee MDPI, Basel, Switzerland. This article is an open access article distributed under the terms and conditions of the Creative Commons Attribution (CC BY) license (http://creativecommons.org/licenses/by/4.0/).

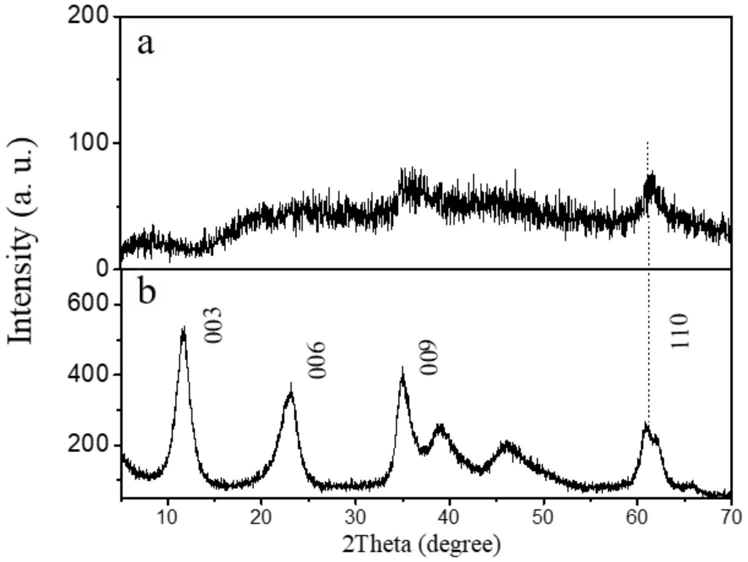

Figure S1. XRD patterns of (**A**) the wet NiFeCo-mono colloid sample, and (**B**) the NiFeCo-mono after drying at 100 degree, respectively.

The disappeared (*00l*) peaks of NiFeCo-mono colloid sample (Figure S1a) indicated the monolayer LDH nanosheets without stacking along *c*-axis. The (110) peak at ~61° indicated the formation of 2D LDH nanosheets. After drying the colloid NiFeCo-mono samples at 100 degree, the obtained NiFeCo-mono powder showed the appearance of (*00l*) peaks, suggesting the stacking LDH structure.

Based on the ICP results, we can obtained the LDH structure with the ratio of Ni:Fe:Co = 2.00 : 1.00 : 0.40

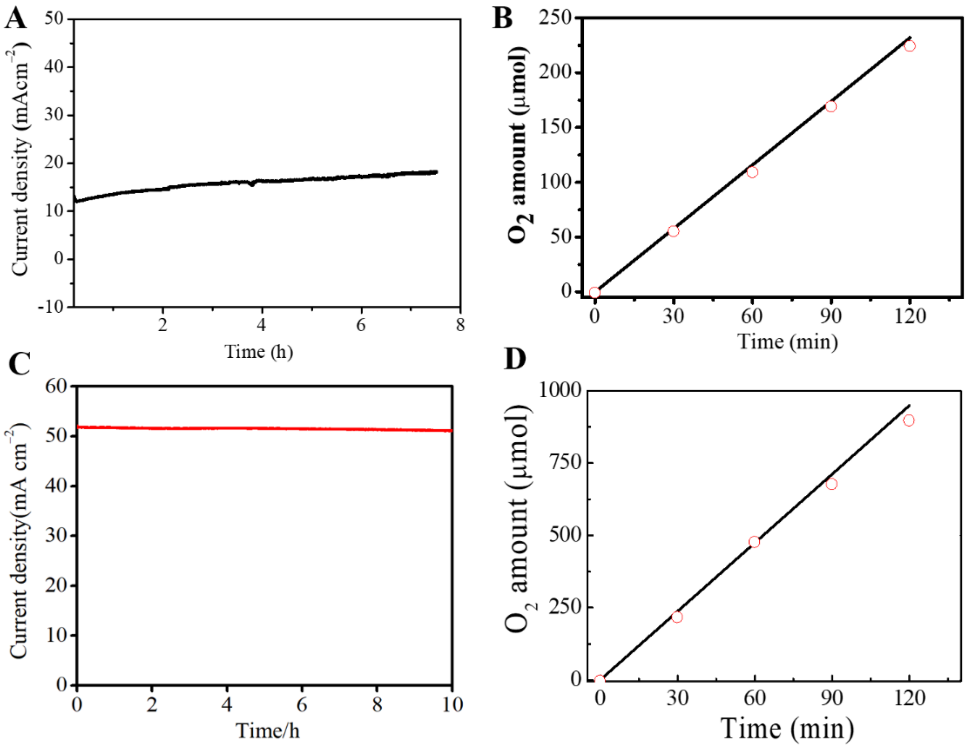

Figure S2. I-t curve and the electrocatalytic efficiency of electrode loaded with NiFeCo-mono at (**A**, **B**) 1.436 V *vs*. RHE and at (**C**, **D**) 1.540 V *vs*. RHE, respectively. (The solid line in (**B**, **D**) means the theoretically expected O_2, and the circle respends to the experimentally tested O_2 from GC with TCD detector).

The Faradic efficiency of the monolayer LDH was defined as the ratio of the evolution amount of O_2 as tested using TCD to that of the theoretically expected O_2 from the water oxidation process. The theoretical amount of O_2 was then calculated by applying the Faraday law, 96485.4 C causes 1 equivalent of reaction, and four mol electrons equal to 1 mol O_2 during OER process.

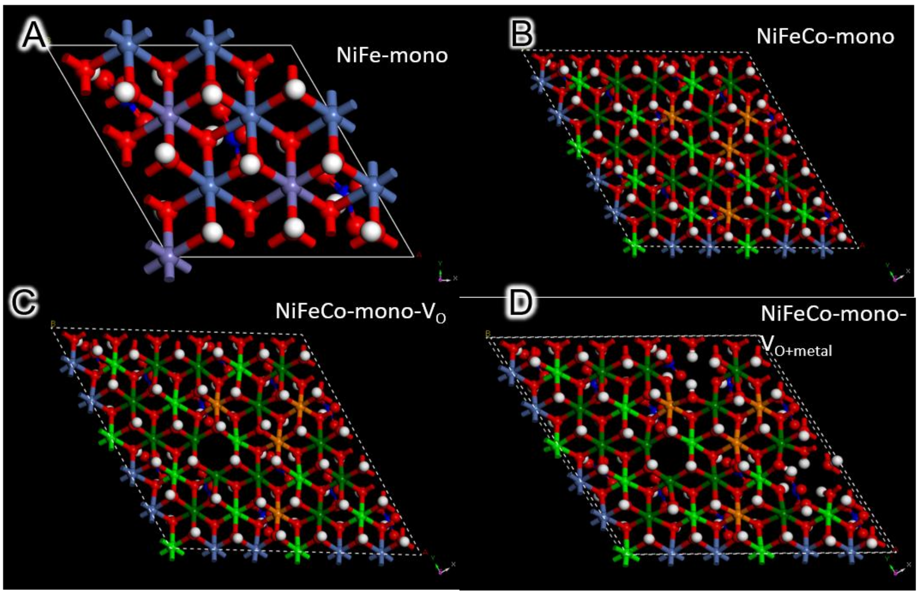

Figure S3. The models of (**A**) NiFe-mono, (**B**) NiFeCo-mono, (**C**) NiFeCo-mono-V_O and (**D**) NiFeCo-mono-$V_{O+metal}$.

Table S1. Fitting results from the EAXFS data of NiFe-mono and NiFeCo-mono.

Sample	Shell	N^a	R[Å][b]	σ^2 [10^{-3} Å^2][c]	S_0^2	R-Factor (10^{-2})
NiFe-mono	Fe–O	5.7	2.00	4.7	1	0.74
	Fe–M	5.3	3.10	6.6		
–			–		–	–
–	Fe–O	5.4	2.00	5.9	0.81	0.54
NiFeCo-mono	Fe–Ni	4.7	3.10	8.5		

[a]N = coordination number; [b]R = distance between absorber and backscatter atoms; [c]σ^2 = Debye-Waller factor.

References

1. Xu, S.-M.; Yan, H.; Wei, M., J.; Band structure engineering of transition-metal-based layered double hydroxides toward photocatalytic oxygen evolution from water: a theoretical−experimental combination study. *Phys. Chem. C* **2017,** *121*, 2683-2695. doi: 10.1021/acs.jpcc.6b10159
